# *Bunias erucago* L.: Glucosinolate Profile and *In Vitro* Biological Potential

**DOI:** 10.3390/molecules24040741

**Published:** 2019-02-19

**Authors:** Ivica Blažević, Azra Đulović, Vedrana Čikeš Čulić, Franko Burčul, Ivica Ljubenkov, Mirko Ruščić, Ivana Generalić Mekinić

**Affiliations:** 1 Department of Organic Chemistry, Faculty of Chemistry and Technology, University of Split, Ruđera Boškovića 35, 21000 Split, Croatia; azra@ktf-split.hr; 2 School of Medicine, University of Split, Šoltanska 2, 21000 Split, Croatia; vedrana.cikes.culic@mefst.hr; 3 Department of Analytical Chemistry, Faculty of Chemistry and Technology, University of Split, Ruđera Boškovića 35, 21000 Split, Croatia; franko@ktf-split.hr; 4 Department of Chemistry, University of Split, Faculty of Science, Ruđera Boškovića 33, 21000 Split, Croatia; ivica.ljubenkov@pmfst.hr; 5 Department of Biology, University of Split, Faculty of Science, Ruđera Boškovića 33, 21000 Split, Croatia; mrus@pmfst.hr; 6 Department of Food Technology and Biotechnology, Faculty of Chemistry and Technology, University of Split, Ruđera Boškovića 35, 21000 Split, Croatia; gene@ktf-split.hr

**Keywords:** *Bunias erucago* L., glucosinolates, desulfoglucosinolates, isothiocyanates, nitriles, anti-oxidation, cholinesterase inhibition, cytotoxic activity

## Abstract

*Bunias erucago* belongs to the Brassicaceae family, which represents a forgotten crop of the Euro-Mediterranean area. The aim of the present study was to determine the glucosinolate profile in different plant parts and biological properties (antioxidant, anticholinesterase, and cytotoxic activities) of the isolates containing glucosinolate breakdown products. The chemical profiles were determined by using HPLC-PDA-MS/MS of desulfoglucosinolates and GC-MS of glucosinolate degradation products. The analysis of *B. erucago* showed the presence of seven glucosinolates: gluconapin (**1**), glucoraphasatin (**2**), glucoraphenin (**3**), glucoerucin (**4**), glucoraphanin (**5**), glucotropaeolin (**6**), and glucosinalbin (**7**). The total glucosinolate content ranged from 7.0 to 14.6 µmol/g of dry weight, with the major glucosinolate glucosinalbin in all parts. The antioxidant activity of all volatile isolates was not notable. At a tested concentration of 227 μg/mL, flower hydro-distillate (FH) showed good AChE inhibition, i.e., 40.9%, while root hydro-distillate (RH) had good activity against BChE, i.e., 54.3%. FH showed the best activity against both tested human bladder cancer cell lines, i.e., against T24 after 72 h, which have IC_50_ of 16.0 μg/mL, and against TCCSUP after 48 h with IC_50_ of 7.8 μg/mL, and can be considered as highly active. On the other hand, RH showed weak activity against tested cancer cells.

## 1. Introduction

*Bunias* is the genus of Brassicaceae family that includes only three accepted species, *B. erucago* L. (crested warty cabbage, corn rocket), *B. orientalis* L. (Turkish warty cabbage), and *B. cochlearioides* Murray. *B. erucago* is a plant native to parts of Southern Europe, Mediterranean Africa, and Turkey [1,2]. In some areas, it is cultivated on a small scale, since it is a key ingredient of different traditional dishes, but it is mostly collected in the wild and is considered a “neglected crop” [1]. Leaves, young stems, and roots are edible and usually eaten raw in salads [3,4] or as a cooked vegetable [5,6]. “Pazija,” (a Turkish name for beet (Beta)) or “pakoleć,” is traditional food prepared as a mixture of wild vegetables boiled in a mix where the dominant species is *B. erucago* [7].

Plants of the Brassicaceae family are highly characterized by the presence of glucosinolates (GSLs) and β-thioglucoside-*N*-hydroxysulfates that contain variable side chains. More than 130 different structures have been identified, which originate from different known amino acids (Ala, Glu, Val, Leu, Ile, Met, SeMet, Trp, Phe, Tyr), and some from uncertain precursors [8]. Their concentrations and profiles vary between plant species and within varieties [9]. Various volatile compounds can be formed by GSL degradation such as mostly isothiocyanates (ITCs) and nitriles. Their presence is usually responsible for designated biological activities. The reports of GSLs breakdown products from this genus are available only for *B. orientalis*, while GSLs of *B. erucago* were not previously reported. Daxenbichler et al. reported the presence of GSLs by their breakdown products: 4-(methylsulfinyl)butyl ITC (originated from glucoraphanin), 4-(methylsulfinyl)but-3-enyl ITC (originated from glucoraphenin), SCN^−^ (released by unstable hydrolysis products such as *p*-hydroxybenzyl ITC and the indolyl GSLs), and isopropyl ITC (originated from glucoputranjivin) [10,11]. Bennet et al. reported the results of LC-MS analysis of different plant parts, which revealed thio-functionalized GSLs such as glucoerucin, glucoraphanin, glucoraphenin, and aromatic ones such as glucosinalbin, 4-hydroxyglucobrassicin, and 4-methoxyglucobrassicin [12,13]. Recently, Maurizi et al. reported that, of the present lipids (0.22 g/100 g edible weight), the main constituents were polyunsaturated fatty acids (71.8%) such as α-linolenic, palmitic, and linoleic acids. In addition, the presence of dietary fibers, minerals (K, Na, Ca, Mg, Fe, P), polyphenols, α-tocopherol, β-carotene, and ascorbic acid, which provide beneficial effects for human health, were determined [14].

In this study, chemical composition of different plant parts of *B. erucago* (flower, leaf and stem, root) was investigated. However, qualitative and quantitative analysis of GSLs using HPLC-PDA-MS/MS analysis of desulfoGSLs and GC-MS analysis of their breakdown volatiles was previously not reported. The volatile isolates were obtained by hydro-distillation in a Clevenger type apparatus and CH_2_Cl_2_ extraction after autolysis. To assess biological potential of *B. erucago*, we investigated its antioxidant activity using DPPH and FRAP assays, cholinesterase inhibitory activity against acetylcholinesterase and butyrylcholinesterase by using the Ellman method, and the cytotoxic activity against two human bladder cancer cell lines (T24 and TCCSUP) by using the MTT method. 

## 2. Results and Discussion

### 2.1. Glucosinolates and Volatile Constituents

**Figure 1:**
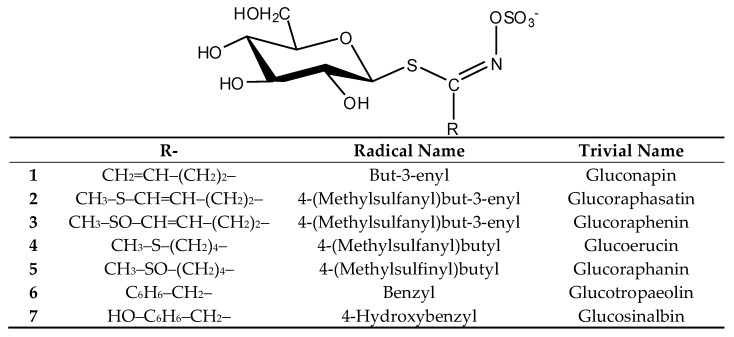
Glucosinolates identified in different parts of *B. erucago*.

*Bunias erucago* was divided into three parts—flower, leaf and stem, and root—and analyzed for the presence of GSLs by HPLC-PDA-MS/MS analyses of their desulfo counterparts (Table 1 and Figures S1–S3) and GC-MS of their breakdown products obtained after hydro-distillation, i.e., extraction (Table 2). GSLs were quantified by HPLC analyses of desulfoglucosinolates (DS-GSLs), according to the ISO 9167-1 method (Table 1). The HPLC-PDA-MS/MS and GC-MS analyses revealed the presence of seven GSLs namely gluconapin (**1**), glucoraphasatin (**2**), glucoraphenin (**3**), glucoerucin (**4**), glucoraphanin (**5**), glucotropaeolin (**6**), and glucosinalbin (**7**). Details about GSLs’ and volatiles’ identification are given in the Materials and Methods Section 3.3.4. The structures of the corresponding GSLs are shown in Figure 1. 

The most abundant peak observed at *t*_R_ = 11.3 min in all the extracts analysed was identified as desulfoglucosinalbin (**d7**), which has 5.6, 6.8, and 8.3 μmol/g of dry weight (dw) in leaf and stem, flower and root, respectively. The degradation products of seven were not detected by GC-MS analysis. 4-Hydroxybenzyl ITC, degradation product of glucosinalbin, is known to be unstable since it tends to react with water and is prone to fast hydrolysis, which makes it generally undetectable by GC-MS [15]. Volatiles that may derive from the same GSL 2-(4-hydroxyphenyl)acetonitrile, or 4-hydroxybenzyl alcohol, were also not detected [16]. Other major peaks, found in flower, leaf and stem, and root extract was desulfoglucoraphenin (**d3**), i.e., 5.7, 1.0, and 1.6 μmol/g dw, respectively. 4-(Methylsulfinyl)but-3-enyl ITC, which is a degradation volatile of **3**, was detected only in CH_2_Cl_2_ extract after autolysis likely due to its thermal instability during hydro-distillation (100 °C). This volatile may degrade into but-3-enyl ITC [17], which was detected in all volatile isolates (0.8%–10.3%). However, but-3-enyl ITC can originate from degradation of **1**, which was found at *t*_R_ = 12.1 min in all parts ranging from 0.2 to 0.6 μmol/g dw. Desulfoglucoraphasatin (**d2**) was detected at *t*_R_ = 19.5 min in flower and root having 1.0 and 2.2 μmol/g dw, respectively. The corresponding degradation products, 5-(methylsulfanyl)pent-4-enenitriles (19.2%) and 4-(methylsulfanyl)but-3-enyl ITC (0.1%), originating from **2**, were also detected in the same plant parts.

Small quantities of other GSLs were also detected including desulfoglucotropaeolin (**d6**) and desulfoglucoerucin (**d4**). Volatiles originating from degradation of **6** and **4** were detected, but in small percentages. Some of the other sulfur compounds that can contribute to the overall biological activity of the isolates were dimethyltrisulfide and dimethyltetrasulfide, which can originate from the cysteinsulfoxide-methiin and 3-methylsulfanylpropanal. Investigated volatile isolates also contained free volatile compounds (without sulfur and/or nitrogen) in very high percentages including hexadecanoic acid (6.1%–53.5%), 6,10,14-trimethylpentadecan-2-one (1.9%–28.2%), and tetradecanoic acid (3.9%–18.3%).

### 2.2. Antioxidative and Anticholinesterase Activity

Hydro-distillates obtained from the *B. erucago* flower and root were tested for their anti-oxidative activity using 2,2-diphenyl-1-picrylhydrazyl (DPPH) and Ferric Reducing Anti-Oxidative Power (FRAP) methods. In the DPPH assay, free radical scavenging ability and hydrogen-donating ability of samples was determined by using stable free radical DPPH, while, in the FRAP assay, antioxidants are evaluated as reductants of Fe^3+^ to Fe^2+^, which is chelated by 2,4,6-tri(2-pyridyl)-s-triazine (TPTZ) to form a Fe^2+^-TPTZ complex. Although the overall antioxidant capacity of the samples was not remarkable. According to the obtained results, it can be seen that the flower hydro-distillate (FH) showed 2-fold higher reducing activity than root hydro-distillate (RH), while there was no notable difference among the free radical scavenging activity of the tested samples. 

Cholinesterase (ChE) inhibitory activity measurements were evaluated using the slightly modified Ellman method. Acetylcholinesterase (AChE) and butyrylcholinesterase (BChE) are key target enzymes in Alzheimer’s disease and ChEs’ inhibitors, which still represent the main pharmacotherapeutic approach in its treatment. Although there are numerous reports on ChE inhibitory activity of various essential oils, reports on the activity of so-called mustard oils are scarce [22,23]. One such rare report includes the AChE inhibitory activity of *Alyssoides utriculata* (L.) Medik seed CH_2_Cl_2_ extract as well as the hydro-distillate of the whole plant, which showed 53.9% and 53.3% inhibition, respectively, at a concentration of 320 μg/mL [24]. The results for essential oils are usually expressed as IC_50_ and ranked as the following: <50 μg/mL excellent, <100 μg/mL very good, and <500 μg/mL good [22]. According to the results obtained at the final concentration of 227 μg/mL, FH showed good AChE inhibition, while RH had good activity against BChE (Table 3).

### 2.3. Cytotoxic Activity

**Figure 2:**
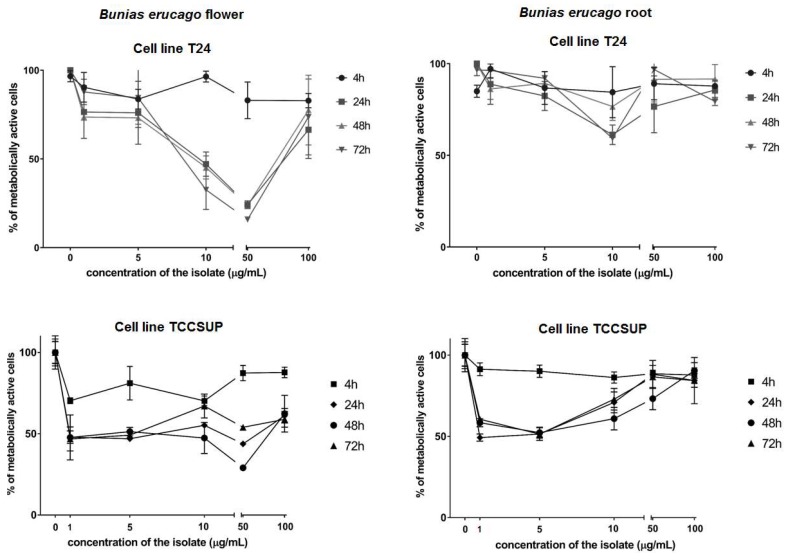
Percentage of metabolically active human bladder cancer cells T24 and TCCSUP after 4, 24, 48, and 72 h of incubation with different concentrations of *B. erucago* hydro-distillate from the flower and roots tested.

The cytotoxic activity was tested against two human bladder cancer cell lines T24 and TCCSUP (Figure 2) and IC_50_ values were calculated (Table 4).

The criteria used to categorize the activity against the tested cell lines was based on IC_50_ values as follows: 20 μg/mL = highly active, 21–200 μg/mL = moderately active, 201–500 μg/mL = weakly active, and >501 μg/mL = inactive [25]. According to the IC_50_, the best cytotoxic activity of flower hydro-distillate (FH) against T24 cells was shown after 72 h with IC_50_ of 16.0 µg/mL, while the best activity against TCCSUP cells was shown after 48 h with IC_50_ of 7.8 µg/mL. These activities can be considered as highly active. On the other hand, root hydro-distillate (RH) showed the best activity against T24 cells after 24 h with IC_50_ 267.5 µg/mL, while the best activity against TCCSUP cells was shown after 48 h with IC_50_ of 258.3 µg/mL. These activities can be considered as weakly active. Based on the GC-MS analysis, it can be suggested that nitriles, present in a higher amount in flower volatiles, are responsible for notable biological activities of FH.

## 3. Material and Methods

### 3.1. Plant Material

*Bunias erucago* L. (flower, leaf and stem, and root) was collected on Island of Brač (near Split, Central Dalmatia, Croatia) (43°21′19.8″ N, 16°36′15.7″ E) during the spring flowering phenological stage in April 2017 from wild-growing populations over the area of 10 m^2^. The primary samples were composed of more than 10 increments and the whole plant material was compiled and dried. A local botanist, Assoc. Prof. Mirko Ruščić, from the Faculty of Natural Sciences, University of Split, confirmed the botanical identity of the plant material. The voucher specimens (ZOKBE001) are deposited at the Department of Organic Chemistry, Faculty of Chemistry and Technology, Split, Croatia.

### 3.2. Reagents

Myrosinase, sinigrin, and rapeseed standard reference material of known glucosinolate composition were obtained from Sigma Aldrich (Steinheim, Germany). Glucoraphanin and glucotropaeolin were obtained from Phytoplan (Heidelberg, Germany), while sinalbin was isolated from the seeds of *Sinapis alba* L., and glucoerucin from *Eruca vesicaria* (L.) Cav. All other chemicals and reagents were of an analytical grade. Cancer cell lines (human bladder cancer cell line T24 and TCCSUP) were cultured in a humidified atmosphere with 5% CO_2_ at 37 °C, in a Dulbecco’s modified Eagle’s medium (DMEM, EuroClone, Milano, Italy) containing 4.5 g/L glucose, 10% fetal bovine serum (FBS), and 1% antibiotics (Penicillin Streptomycin, EuroClone, Milano, Italy).

### 3.3. Isolation and Chemical Analysis

#### 3.3.1. Isolation

Flower, leaf and stem, and root were ground to a fine powder. Samples of ca. 100 mg were extracted for 5 min at 80 °C in 2 × 1 mL MetOH/H_2_O (70:30 *v*/*v*) to inactivate the endogenous myrosinase using a GLH 850 homogenizer (OMNI International, Kennesaw, GA, USA) and then centrifuged for 10 min. Supernatants were combined to reach a final volume of 2 mL. Each extract (1 mL) was loaded onto a mini-column filled with 0.6 mL of DEAE-Sephadex A-25 anion-exchange resin (GE Healthcare, Pittsburgh, PA, USA) conditioned with 25 mM acetate buffer (pH 5.6). After washing the columns with 2 mL of 70% MetOH for the removal of nonpolar compounds and 1 mL of ultrapure water, optimal conditions for desulfation were created by adding 2 mL of buffer solution. In addition, 20 μL (0.35 U/mL) of purified sulfatase [26] was loaded onto the mini-columns as well as 50 μL of buffer in order to distribute the sulfatase equally and were left to stand overnight at 30 °C. The DS-GSLs were then eluted with 1.5 mL of ultra-pure H_2_O and the samples were stored at −20 °C until further analysis.

#### 3.3.2. HPLC-PDA-MS/MS Analysis and Quantification of Desulfoglucosinolates 

Analysis was performed by injecting a 10 µL aliquot of the solution of desulfated crude extract into a high-performance liquid chromatograph LCMS-8050 (Shimadzu Europe GmbH, Duisburg, Germany) equipped with a quaternary pump, automatic injector, photodiode-array detector (wavelength range 190–600 nm), a vacuum degasser, and a ZORBAX Eclipse XDB-C18 column (250 mm × 4.0 mm, 5 µm particle size, Agilent, Santa Clara, CA, USA). A gradient consisting of solvent A (50 μM NaCl in H_2_O) and solvent B (acetonitrile:H_2_O = 30:70 *v*/*v*) was applied at a flow rate of 0.8 mL/min as follows: 0.5 min 96% A and 4% B, 28 min 14% A and 86% B, 4.0 min 14% A and 86% B, 2.0 min 5% A and 95% B, 13.0 min 5% A and 95% B, 1.0 min 96% A and 4% B, and 8.0 min 96% A and 4% B. The column temperature was 30 °C. After each run, the initial mobile phase conditions were set and the system was allowed to equilibrate for 5 min. The electrospray interface was a standard ESI source operating with a capillary voltage of 4 kV and temperature of 350 °C. The system was operated in the positive ion electrospray mode. Nitrogen was used as nebulizing gas at a flow rate of 3 L/min and as drying gas at a flow rate of 10 L/min.

The amount of GSLs was quantified by using an external calibration curve (y = 3102.4910x, R^2^ = 0.9938, LOD = 1.67 µM, LOQ = 5.01 µM) of pure DS-SIN solution (concentrations used: 13.56, 27.13, 54.25, 162.75, 217.00, 542.00, and 651.00 µM) and response factors (RFs) for each individual DS-GSL (1.11 for **d1,** 0.4 for **d2**, 0.9 for **d3**, 0.9 for **d4**, 0.9 for **d5**, 0.95 for **d6**, and 0.5 for **d7**) [26,27,28]. The column and all the conditions of analysis were the same as those previously used for identification. Detection was carried out by HPLC-PDA at 227 nm (Figures S1–S3).

#### 3.3.3. Isolation and GC-MS Analysis of Volatiles 

The volatiles were isolated by hydro-distillation using whole dried plant (ca. 20 g) and extraction using different plant parts (10 g). Hydro-distillation was performed in the Clevenger apparatus (Deotto Lab, Zagreb, Croatia) for 2.5 h, while extraction was done by CH_2_Cl_2_ after hydrolysis by myrosinase (1–2 units) for 24 h at 27 °C [29]. All the isolates were analyzed by GC-MS using the VF-5MS column (30 m × 0.25 mm i.d., coating thickness 0.25 μm, Palo Alto, CA, USA). VF-5MS column temperature was programmed at 60 °C isothermal for 3 min, and then increased to 246 °C at a rate of 3 °C/min and held isothermal for 25 min with conditions, as described previously [30]. The analyses were carried out in duplicate.

#### 3.3.4. Methods of Identification

Glucoraphenin, gluconapin, glucotropaeolin, glucoerucin, and sinalbin were identified by comparison of *t*_R_, UV, and mass spectra with obtained standards. Desulfo-glucosinolates (M_DS_ + Na) were monitored based on the following MRM transitions: desulfogluconapin (**d1**), 316,10 > 219, 185, desulfoglucoerucin (**d4**), 364,10 > 219, 202, 185, desulfoglucoraphanin (**d5**), 380,10 > 316, 218, 200, 184, 168, glucotropaeolin (**d6**), 352,10 > 219, 185, glucosinalbin (**d7**) 368 > 219, 206, 185 [31], while the SIM mode (M_DS_ + Na) was used for desulfoglucoraphasatin (**d2**), 362 and desulfoglucoraphenin (**d3**), 378. Individual peaks of volatile compounds were identified by comparing their retention indices and mass spectra with those of authentic samples as well as by computer matching against the Wiley 7 spectral database and comparison of the mass spectra with literature data [18,19,20,21]. The percentages in Table 1 were calculated as the mean value of component percentages on the VF-5MS column for an analyses run in duplicate.

### 3.4. Biological Activities Assays

All spectrophotometric assays (DPPH, FRAP, AChE/BChE, and MTT) were performed on a Tecan Microplate Reader, model Sunrise (Tecan Group Ltd., Männedorf, Switzerland). 

#### 3.4.1. Antioxidant Activity 

For the antioxidant activity evaluation, two methods were used: 2,2-diphenyl-1-picrylhydrazyl (DPPH), and Ferric Reducing/Antioxidant Power (FRAP) assay.

**DPPH assay:** DPPH scavenging ability of the samples was measured, according to the procedure previously reported [23]. DPPH radical solution was prepared by dissolving DPPH in EtOH to get an initial absorbance of 1.2 ± 0.02. A sample aliquot (10 µL) was added to 290 µL of DPPH solution, stirred, and left to stand at room temperature in the dark. The decrease in absorbance was measured at 517 nm after 60 min. The results for radical scavenging activity are expressed as an inhibition percentage of the DPPH radical (% of inhibition). All determinations were performed in triplicate.

**FRAP assay:** The reducing potential of isothiocyanates was measured as described previously [23]. The working FRAP reagent was prepared by mixing 300 mM acetate buffer, 10 mL TPTZ in 40 mM HCl, and 20 mM FeCl_3_·6H_2_O in proportion of 10:1:1. A sample (10 µL) was added to the freshly prepared working FRAP reagent (300 µL). The absorbance of the blue color complex was recorded against a blank at 593 nm after 4 min. A standard curve was prepared using different concentrations of Fe^2+^, determinations were performed in triplicate, and the results are expressed in µmol Fe^2+^ per liter of extract (µM Fe^2+^). 

#### 3.4.2. Acetylcholinesterase/Butyrylcholinesterase Inhibitory Activity 

AChE/BChE inhibitory activity measurements were carried out by using a slightly modified Ellman method, as described earlier [23]. Reactants (180 µL of 0.1 M phosphate buffer, pH 8), 10 µL of 5,5′-dithio-bis(2-nitrobenzoic acid) at a final concentration of 0.3 mM prepared in 0.1 M phosphate buffer, pH 7, with 0.12 M sodium bicarbonate, 10 µL of sample, 10 µL AChE/BChE solution at a final concentration of 0.03 U/mL were mixed. The reaction was initialized by adding 10 µL of acetyl/butyrilthiocholine iodide at a final concentration of 0.5 mM. ETOH was used as a negative control and non-enzymatic hydrolysis was monitored by measuring two blank runs for each run. First, blank buffer was added instead of the enzyme mixture and, second, blank buffer was added instead of acetyl/butyrilthiocholine iodide. All measurements were done at 409 nm and room temperature for a six-minute period. The results are expressed as a percentage inhibition of enzyme activity.

#### 3.4.3. Cell Proliferation Assay (MTT)

Cells were re-suspended in a diluted solution of trypan blue and counted by a binocular inverted microscope, MOTIC AE30 (Motic, Barcelona, Spain), using the Neubauer chamber (Blaubrand, BRAND GMBH, Wertheim, Germany). The cell number was calculated, according to the formula: number of counted cells × dilution × 10^4^/mL. The cells were then placed in 96-well plates at a density of 11,000 cells/well and incubated overnight. The cells were treated with *B. erucago* flower and root hydro-distillate at concentrations of 1, 5, 10, 50, and 100 µg/mL in a complete medium (in triplicate) for 4, 24, 48, and 72 h. Then, the MTT assay was performed in such a manner that, after the treatment with isolated compounds, the cells were incubated with 0.5 g MTT/L at 37 °C for 2 h. After that, the medium was removed and dimethylsulfoxide (10% DMSO) was added and incubated for another 10 min at 37 °C while shaking. The degree of formazan formation, which is an indicator of living and metabolically active cells, was measured at 570 nm. The data were calculated in relation to the untreated control (100%) from three independent measurements [32]. Cisplatin (50 μg/mL) was used as the positive control. The calculation of IC_50_ values was done using the GraphPad Prism software version 7.0 (San Diego, CA, USA).

## 4. Conclusions

This is the first report of the seven GSLs, which originates from Phe (compounds **6**, **7**) and Met (compounds **1**–**5**) biosynthesis pathways, are present in *B. erucago*. The volatile isolates containing their degradation products showed positive biological activity. Flower hydro-distillate (FH) showed good AChE inhibitory activity, while root hydro-distillate (RH) showed good activity against BChE. Cytotoxic activity of FH against both cancer cell lines was highly active in comparison to weekly active RH. The observed activities could be attributed to the presence of the GSL degradation products. 

Expected degradation products of glucosinalbin (**7**)**,** which is the main GSL in all investigated plant tissues, was not detected by GC-MS analysis. Water solubility and/or the instability due to the high temperature (100 °C) applied during isolation, as well as hydrolysis, were responsible for the loss of these volatile compounds. Thus, although glucosinalbin (**7**) was the main GSL constituent, its degradation products could not contribute to the bioactivity of volatile isolates. Other methods of isolation, by avoiding the water medium and/or high temperatures, should be employed in order to obtain degradation products of glucosinalbin (**7**), which enables the assessment of their contribution to the overall bioactivity.

## Figures and Tables

**Table 1:** Individual concentrations of glucosinolates (μmol/g dw) in *B. erucago* tissues.

Glucosinolate	*t*_R_ (minutes)	Flower	Leaf and Stem	Root
Glucoraphanin (**5**)	8.1	0.3 ± 0.0	0.1 ± 0.0	0.1 ± 0.0
Glucoraphenin (**3**)	8.4	5.7 ± 0.8	1.0 ± 0.2	1.6 ± 0.4
Glucosinalbin (**7**)	11.3	6.8 ± 0.5	5.6 ± 0.9	8.3 ± 1.0
Gluconapin (**1**)	12.1	0.6 ± 0.1	0.2 ± 0.0	0.5 ± 0.1
Glucotropaeolin (**6**)	18.2	0.2 ± 0.0	0.1 ± 0.0	0.2 ± 0.0
Glucoerucin (**4**)	18.9	-	-	tr
Glucoraphasatin (**2**)	19.5	1.0 ± 0.2	-	2.2 ± 0.2
Total (μmol/g dry weight)		14.6 ± 1.6	7.0 ± 1.1	12.9 ± 1.7

dw, dry weight; tr < 0.1 μmol/g dw; -, not detected.

**Table 2:** Glucosinolates, their breakdown products, and other volatiles identified in different parts of *B. erucago* by GC-MS.

			Hydrodistilate			Extract
Glucosinolate	Identified Breakdown Compound	RI	Flower	Leaf and Stem	Root	Whole Plant Material
Gluconapin (**1**)	But-3-enyl ITC ^a,b,c^	993	0.8	2.0	10.3	-
Glucotropaeolin (**6**)	2-Phenylacetonitrile ^a,b^	1178	1.0	tr	-	-
	Benzyl ITC ^a,b^	1394	-	-	tr	-
Glucoraphasatin (**2**)	5-(Methylsulfanyl)pent-4-enenitrile *^,a,b^	1214	14.5	-	1.4	-
	5-(Methylsulfanyl)pent-4-enenitrile *^,a,b^	1251	4.7	-	1.0	-
	4-(Methylsulfanyl)but-3-enyl ITC ^a,b,c^	1440	0.1	-	2.4	-
Glucoerucin (**4**)	4-(Methylsulfanyl)pentanenitrile ^a,b,c^	1233	2.3	-	-	-
	4-(Methylsulfanyl)butyl ITC (erucin) ^a,c^	1457	-	-	1.1	-
Glucoraphenin (**3**)	4-(Methylsulfinyl)but-3-enyl ITC ^a,c^	1817	-	-	-	88.3
**Other Volatiles**						
1,2-Dimethylbenzene ^b^		874	3.9	0.8	4.0	-
3-Methylsulfanylpropanal ^b^		919	-	-	0.3	-
Dimethyltrisulfide ^a,b^		981	tr	-	9.4	-
Benzeneacetaldehyde ^a,b^		1063	0.5	1.1	1.3	-
(*E*)-Non-2-en-1-ol ^a,b^		1113	1.3	0.8	0.5	-
Dimethyltetrasulfide ^a,b^		1235	-	-	0.3	-
4-Vinyl-2-methoxyphenol ^a,b^		1353	0.7	0.8	tr	-
(*E*)-β-Ionone ^a,b^		1492	1.8	-	-	-
Tetradecanoic acid ^a,b^		1828	3.9	18.3	6.7	-
6,10,14-Trimethylpentadecan-2-one ^a,b^		1854	28.2	25.5	1.9	-
Hexadecanoic acid ^a,b^		2016	24.6	39.8	53.5	6.1
Total sum (%)			88.3	89.1	94.1	94.4

RI, Retention indices determined on a VF-5MS capillary column. -, not detected. tr, traces. *, correct isomer is not identified. ITC, isothiocyanate. ^a^ Compound identified by mass spectra and RI comparison with own library. ^b^ Compound identified by mass spectra comparison with Wiley library. ^c^ Compound identified by mass spectra comparison with literature values [18,19,20,21].

**Table 3:** Antioxidant and cholinesterase inhibitory activity of volatile isolates from *Bunias erucago.*

	Flower Hydro-Distillate (FH)	Root Hydro-Distillate (RH)
**Antioxidant Activity**		
DPPH (Inhibition %)	9.6 ± 0.4	10.2 ± 1.4
FRAP (µM Fe^2+^)	41.7 ± 0.9	24.3 ± 0.1
**Cholinesterase Activity**		
AChE (Inhibition %)	40.9 ± 0.2	13.7 ± 0.2
BChE (Inhibition %)	25.0 ± 0.1	54.3 ± 0.3

DPPH—2,2-Diphenyl-1-picrylhydrazyl. FRAP—Ferric Reducing Antioxidant Power. AChE—acetylcholinesterase inhibitory activity. BChE—butyrylcholinesterase inhibitory activity. All samples were tested in triplicates, at final concentrations for DPPH, FRAP, and AChE/BChE of 166, 161, and 227 μg/mL, respectively.

**Table 4:** Cytotoxic activity of volatile isolates from *Bunias erucago* against two human bladder cancer cell lines (T24, TCCSUP) expressed as IC_50_ values (μg/mL).

	Flower Hydro-Distillate (FH)				Root Hydro-Distillate (RH)			
	4 h	24 h	48 h	72 h	4 h	24 h	48 h	72 h
T24	362.6	21.4	23.8	16.0	517.7	267.5	676.0	366.8
TCCSUP	418.4	15.2	7.8	49.9	519.1	326.0	258.3	316.9

## Supplementary Materials

The following are available online, Figure S1: HPLC-PDA chromatograms of the desulfoglucosinolates isolated from flowers of *Bunias erucago* L.: **1**—gluconapin, **2**—glucoraphasatin, **3**—glucoraphenin, **5**—glucoraphanin, **6**—glucotropaeolin, **7**—glucosinalbin. Figure S2: HPLC-PDA chromatograms of the desulfoglucosinolates isolated from leaves and stems of *Bunias erucago* L.: **1**—gluconapin, **3**—glucoraphenin, **5**—glucoraphanin, **6**—glucotropaeolin, **7**—glucosinalbin. Figure S3: HPLC-PDA chromatograms of the desulfoglucosinolates isolated from roots of *Bunias erucago* L.: **1**—gluconapin, **2**—glucoraphasatin, **3**—glucoraphenin, **4**—glucoerucin, **5**—glucoraphanin, **6**—glucotropaeolin, **7**—glucosinalbin.
