# Supplementary material for: Bunias erucago L.: Glucosinolate Profile and In Vitro Biological Potential
Source: Molecules. 2019 Feb 19;24(4):741. doi: 10.3390/molecules24040741 (PMC6412829; doi:10.3390/molecules24040741)
Supplement: Supplementary file 1 [file molecules-24-00741-s001.pdf]

molecules

MDPI

Supplementary Material

Bunias erucago L.: Glucosinolate Profile and In vitro Biological Potential

Ivica Blažević [1,*], Azra Đulović [1], Vedrana Čikeš Čulić [2], Franko Burčul [3], Ivica Ljubenkov [4], Mirko Ruščić [5] and Ivana Generalić Mekinić [6]

[1] Department of Organic Chemistry, Faculty of Chemistry and Technology, University of Split, Ruđera Boškovića 35, 21000 Split, Croatia; azra@ktf-split.hr
[2] School of Medicine, University of Split, Šoltanska 2, 21000 Split, Croatia; vedrana.cikes.culic@mefst.hr
[3] Department of Analytical Chemistry, Faculty of Chemistry and Technology, University of Split, Ruđera Boškovića 35, 21000 Split, Croatia; franko@ktf-split.hr
[4] Department of Chemistry, University of Split, Faculty of Science, Ruđera Boškovića 33, 21000 Split, Croatia; ivica.ljubenkov@pmfst.hr
[5] Department of Biology, University of Split, Faculty of Science, Ruđera Boškovića 33, 21000 Split, Croatia; mrus@pmfst.hr
[6] Department of Food Technology and Biotechnology, Faculty of Chemistry and Technology, University of Split, Ruđera Boškovića 35, 21000 Split, Croatia; gene@ktf-split.hr
* Correspondence: blazevic@ktf-split.hr; Tel.:+385-21-329-434

HPLC-PDA chromatograms (at 227 nm)

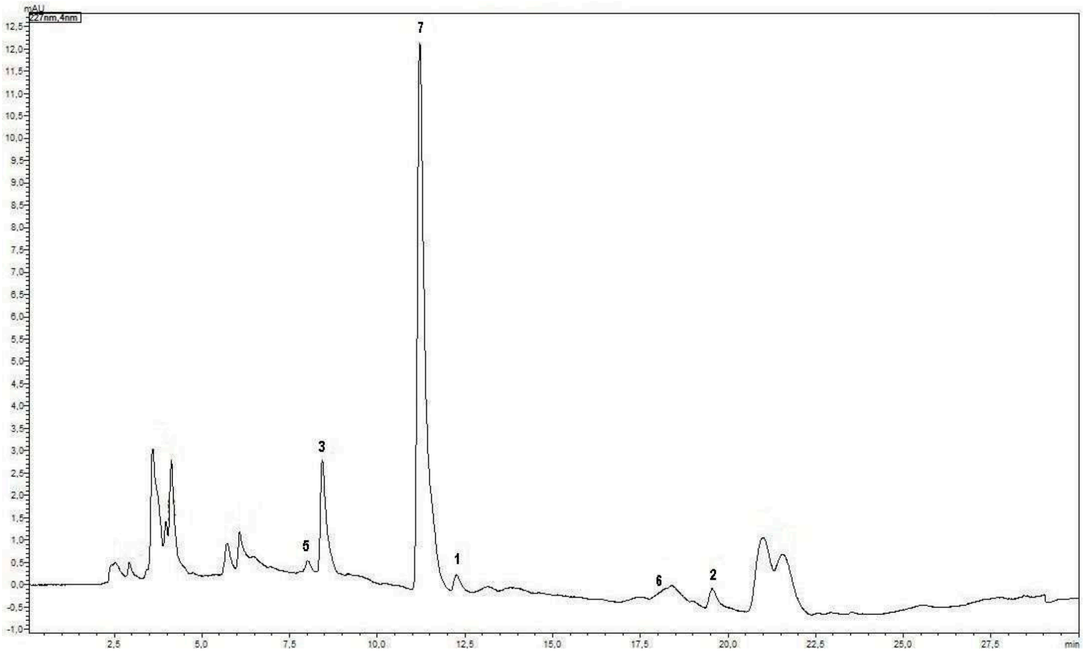

Figure S1. HPLC-PDA chromatograms (at 227 nm) of the desulfoglucosinolates isolated from flowers of Bunias erucago L.: 1—gluconapin; 2—glucoraphasatin; 3—glucoraphenin; 5—glucoraphanin; 6—glucotropaeolin, 7—glucosinalbin.

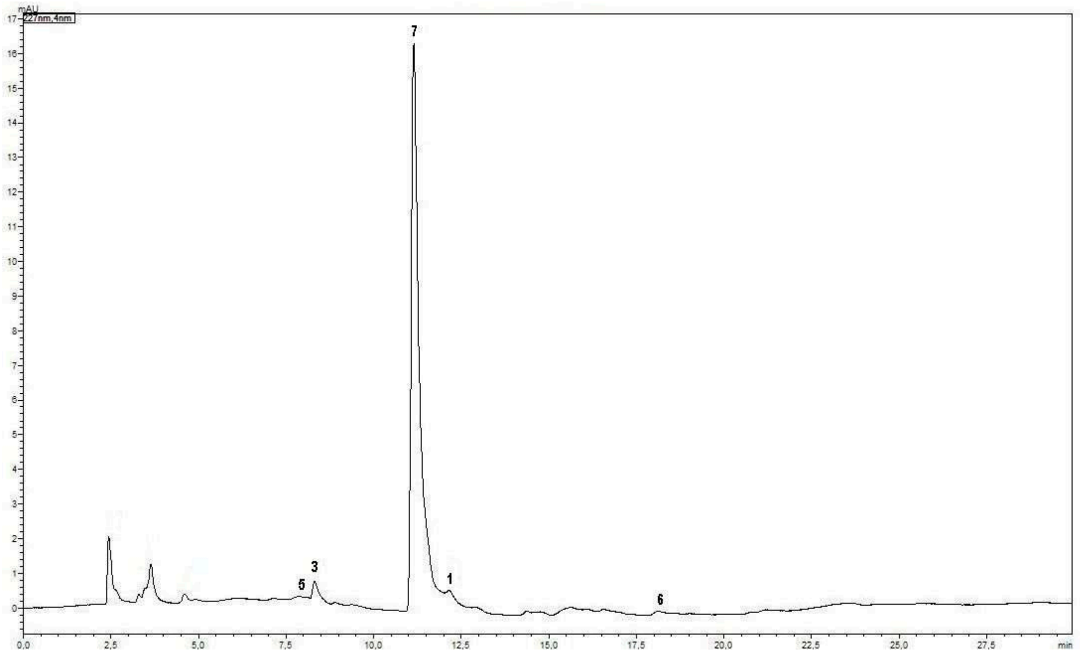

Figure S2. HPLC-PDA chromatograms (at 227 nm) of the desulfoglucosinolates isolated from leaves & stems of Bunias erucago L.: 1—gluconapin; 3—glucoraphenin; 5—glucoraphanin; 6—glucotropaeolin, 7—glucosinalbin.

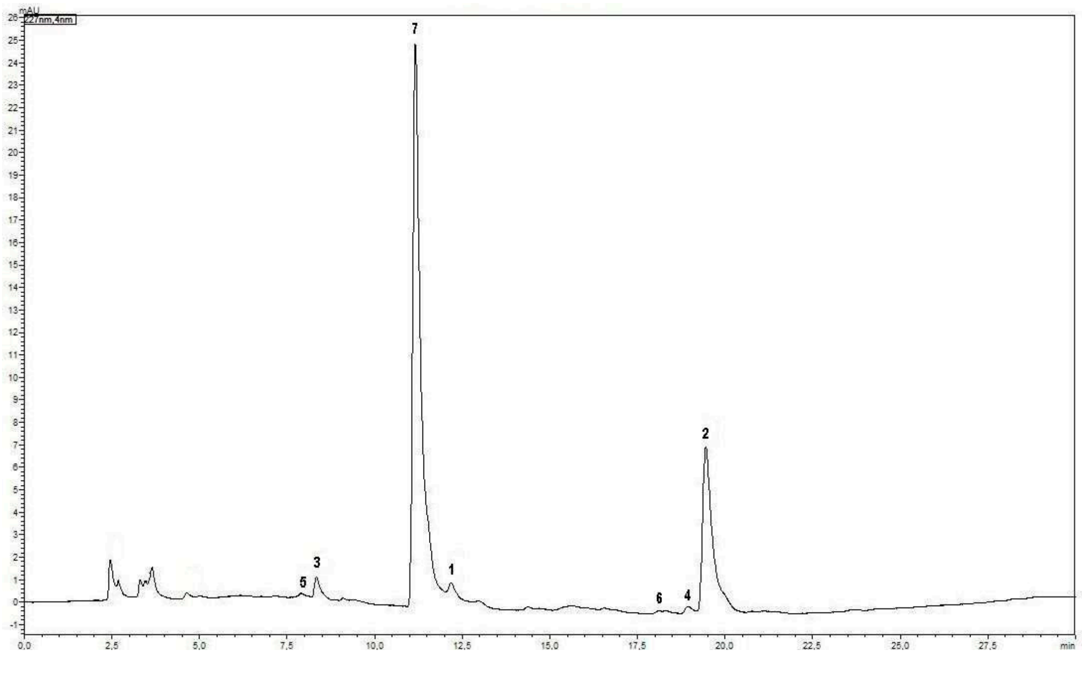

Figure S3. HPLC-PDA chromatograms (at 227 nm) of the desulfoglucosinolates isolated from roots of Bunias erucago L.: 1—gluconapin; 2—glucoraphasatin; 3—glucoraphenin; 4—glucoerucin; 5—glucoraphanin; 6—glucotropaeolin, 7—glucosinalbin.
